# Large Animal Models of Breast Cancer

**DOI:** 10.3389/fonc.2022.788038

**Published:** 2022-02-04

**Authors:** Pinaki Mondal, Katie L. Bailey, Sara B. Cartwright, Vimla Band, Mark A. Carlson

**Affiliations:** ^1^ Department of Surgery, University of Nebraska Medical Center, Omaha, NE, United States; ^2^ Department of Surgery, VA Medical Center, Omaha, NE, United States; ^3^ Department of Genetics, Cell Biology and Anatomy, University of Nebraska Medical Center, Omaha, NE, United States; ^4^ Fred & Pamela Buffett Cancer Center, University of Nebraska Medical Center, Omaha, NE, United States; ^5^ Center for Advanced Surgical Technology, University of Nebraska Medical Center, Omaha, NE, United States

**Keywords:** breast cancer, large animal models, animal models, porcine model, BRCA1, TP53

## Abstract

In this mini review the status, advantages, and disadvantages of large animal modeling of breast cancer (BC) will be discussed. While most older studies of large animal BC models utilized canine and feline subjects, more recently there has been interest in development of porcine BC models, with some early promising results for modeling human disease. Widely used rodent models of BC were briefly reviewed to give context to the work on the large animal BC models. Availability of large animal BC models could provide additional tools for BC research, including availability of human-sized subjects and BC models with greater biologic relevance.

## Background: Breast Cancer Burden

The annual incidence of breast cancer (BC) in women (all ages and races) in the U.S. increased from 0.102% in 1980 to a peak of 0.142% in 1999, and then decreased slightly, plateauing at ~0.131% from 2011-2017 (1). As of 2017, a woman’s lifetime risk of developing BC in the U.S. is 12.9% (1). In 2021, the estimated number of new BC cases in the U.S. will be 281,550 (15.3% of all new cancer cases), with 43,600 estimated deaths (~20 per 100,000 in the general population, or 7% of all cancer deaths, or ~2% of all mortality in the U.S.) (1, 2).

All-stages 5-year survival for BC has improved from 75% in 1975 to 90% in 2016 (1), secondary to earlier diagnosis and more efficacious therapy (3). However, up to 50% of hormone dependent (estrogen/progesterone receptor positive or ER/PR+) BC patients acquire resistance under treatment, and 20% do not even respond to first-line hormone therapies (4). In addition, dormant ER/PR+ tumor cells can become reactivated and can cause disease relapse (5). Almost 50% of ER/PR+ BC tumors are also positive for Erb-B2 Receptor Tyrosine Kinase 2 (ERBB2), commonly referred to as HER2/NEU (human epidermal growth factor receptor 2) (6). While trastuzumab (a monoclonal antibody therapy targeting HER2) is standard treatment for HER2+ BC, almost all patients with metastatic HER2 + BC eventually develop treatment resistance (7, 8).

Survival with triple-negative BC (TNBC; minimal/nil expression of the estrogen receptor, progesterone receptor, and HER2), which accounts for 10-20% of all BC (9, 10), is 10, 20, and 30% lower at stages 2, 3, and 4, respectively, compared to non-TNBC (11). So, there remain a need for improved management of both receptor-positive BC and TNBC. The availability of validated, tractable large animal models that faithfully represent both (1) hormone and growth factor dependent human BC and (2) TNBC should allow development and testing of technologies and treatments that would not be possible in murine models, with resultant data that would be predictive of human tumor response.

## Large Animal Models of BC

### Justification for and Advantages of a Large Animal Model of BC

**Figure 1:**
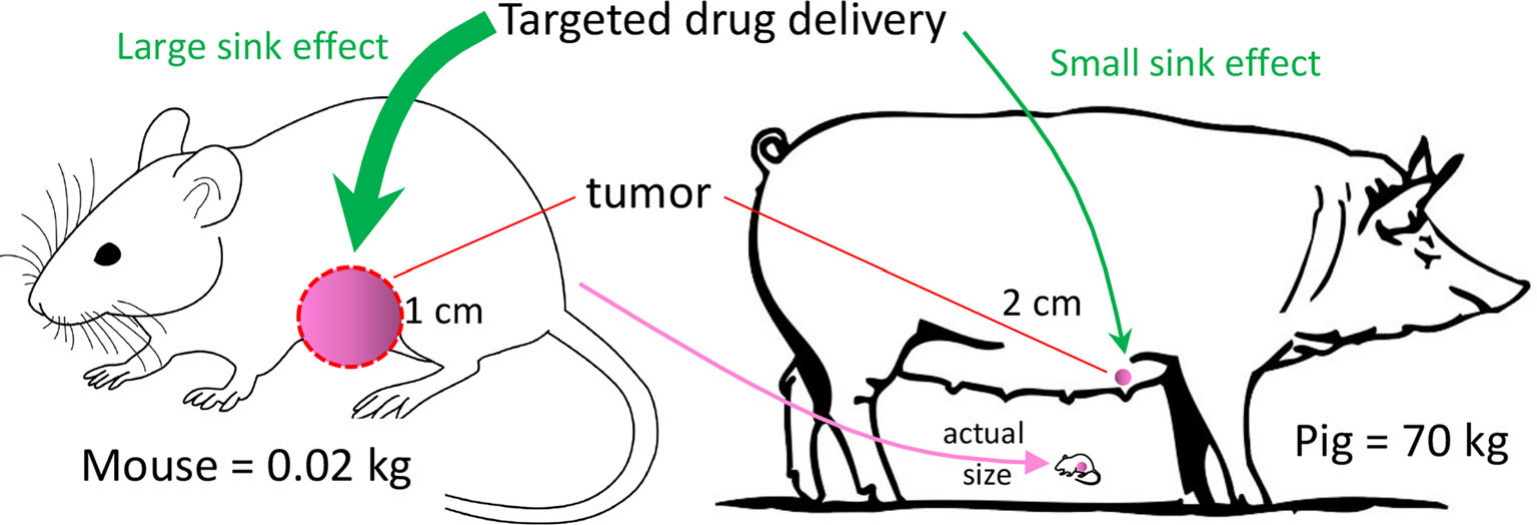
Tumor Sink. In this example, murine tumor:body mass ratio is 400x the porcine ratio, even though murine tumor mass is only ~12% that of the porcine tumor. If a targeted drug preferentially concentrates in the tumor (i.e., tumor sink), then a given drug dose in the mouse may have a relatively high concentration in the tumor with respect to the plasma. The effect of tumor sink on plasma concentration at the same dose in the pig would be negligible because of the large body size. Thus, the same dose in the pig would produce a higher plasma concentration, possibly producing greater systemic toxicity than in the mouse.

Breast cancer, and TNBC in particular, can be modeled with many of the murine and other small animal models. However, none of these models can overcome the limitation of inadequate subject size of small animals. Development of some diagnostic and interventional technologies require a human-sized model to understand how clinically relevant tumor size and tissue thickness affect the performance of the experimental technology, such as with three-dimensional scanning or with a tissue-ablation device. In addition, pharmacokinetic parameters (including absorption, distribution, metabolism, and excretion) can be vastly different between a 20 g mouse and a 70 kg patient (12). Testing the effect of an infused chemical entity (e.g., a novel anti-tumor agent) in a murine tumor model may result in an inaccurate conclusion secondary to these pharmacokinetic differences (**Figure 1**).

With regard to drug distribution, tumor burden in a 20 gram mouse with a 1-cm tumor is ~400-fold greater (mass:mass) than in a 70 kg subject with a 2-cm tumor, as diagramed in **Figure 1**. If the tumor acts as a sink for a candidate anti-tumor drug, then the tumor’s ability to decrease the drug’s plasma concentration would be much greater in the mouse. A consequence of this tumor sink effect (13) would be a gross underestimation of the drug’s toxicity from murine data. Alternatively, if the drug penetrates poorly into dense tumor stroma (14), then testing in a ~1 cm murine tumor may overestimate the drug’s anti-tumor efficacy, as opposed to testing in a large (≥4 cm) tumor. These drug distribution issues in a murine model could be minimized with a large animal model (e.g., a 70 kg pig) with clinically relevant tumor size. So, a primary justification for a large animal BC model would be its ability to replicate human tumor dimension and human PK parameters. Other advantages of large animal BC models (and large animal cancer models in general) have been listed in **Table 1**.

**Table 1:** Advantages of large animal cancer models.

**1**	Large animals can be human-sized, with analogous physiology
**2**	Genotype-phenotype relationships in large animals tend replicate those in humans better than mice do
**3**	Large animal tumors can have human-relevant mass, stroma, and vasculature
**4**	Large animal pharmacokinetics can be similar to humans
**5**	Large animals can undergo serial phlebotomy and multiple survival surgeries
**6**	Cellular and molecular tools normally reserved for mice are increasingly available for some large animals (e.g., pigs)
**7**	Medical devices designed for humans (e.g., endoscopes, surgical instruments, CT scanners) can be used on large animals; conversely, devices developed in large animals can be readily transitioned to humans

## Current Status: Large Animal Models

### Overview

Most of the older work on large animal modeling of BC has focused on feline and canine subjects (**Table 2**). Of note, non-human primates (NHPs) have not been commonly utilized in BC or other cancer research. Recently, developmental work on porcine BC models has emerging, with promising data from both orthotopic implantation strategies and genetic editing. The current status of large animal BC models has been summarized in **Table 2**.

**Table 2:** Current status of large animal models for BC.

Name	Spontaneous BC	Tumor Cell injection	BC Organoid	Morphological, histological, and molecular similarities with human	Genetic model
**Feline**	Yes (15).	BC with metastasis (in nude mice and in feline) (16, 17).	Tumorigenic, Radioresistant, Chemoresistant (18, 19).	Similar in all (15, 20, 21).	Spontaneous model (15).
**Canine**	Yes, with metastasis (22).	BC in nude mice (23).	Normal and tumor organoids (24).	Similar mutations, pathways, histology (25–27).	Spontaneous model (22).
**NHP**	Not commonly used as a model.		*Ex vivo* model (28, 29).	Not commonly used a model.	
**Porcine**	Not known. or rare (30).	BC in nude mice (31, 32).	Normal and transformed organoids (31, 33).	Similar histology, molecular profiles (34).	Preliminary, under development.

### Feline BC Models

Feline mammary carcinomas (FMCs) are the third most common type of cancer in cats (35). *In utero* implantation of allogeneic mammary cancer cell lines into fetal cats (*Felis catus*) produced tumor at the injection site, followed by widespread metastasis after 6-10 weeks (16, 17). A nude mouse model of FMC demonstrated metastatic potential (bone, kidney, brain, lung, and liver; i.e., common sites of metastasis in human breast cancer) after injection to the primary site (36). Cancer stem cell-like populations in FMCs can form mammospheres (organoids) and are tumorigenic, radioresistant, and chemoresistant (18, 19). Mammospheres can be used a model to study feline breast cancer. Morphological, histological, and molecular to similarities between FMCs and human breast cancer have been described and discussed (15, 20, 21).

### Canine BC Models

Similar to cats, canine mammary tumors (CMTs) occur spontaneously and are the second most common cancer in dogs (37). Development of CMTs is hormone dependent and showed dysregulated expression of BRCA1, BRCA2 and TP53 (25–27), analogous to human BC. Transcriptional analysis of CMTs demonstrated pathways that are active in human cancer, including those involved with cell cycle regulation, apoptotic signaling, immune functions, endoplasmic reticulum stress, angiogenesis, and cell migration (38). Approximately 25% of the genetic alterations in metastatic CMTs were associated with human mammary cancer (39). In addition, canine spontaneous mammary DCIS and invasive cancer shows similar histologic and molecular characteristics with DCIS and invasive cancer in humans (40). Spontaneous CMTs also can metastasize to lymph nodes and lung (22). HER2 overexpression in CMTs is controversial (41). Benign tumors and mesenchymal tumors are more prevalent in CMTs; the latter are rare with human breast cancer (42, 43).

Canine inflammatory BC cells were able to generate tumors in nude mice both ectopically and orthotopically (23). No morphological differences in the tumors were noted when human inflammatory BC cells were injected instead of canine cancer cells (23). There are several other canine BC cell lines that were characterized and used in development for BC in nude mice (44–47). A newly developed canine TNBC cell line, B-CMT, also showed BC in nude mice after 14 days of orthotopic inoculation (48). Canine normal mammary tissue and BC tissue were used to develop organoids, which showed multi lineage potential and existence of different population of self-renewing stem cells (24). These canine normal and BC organoids will serve as valuable models for future research with multiple species.

### Non-Human Primate BC Models

Mammary gland tissue from common marmoset and rhesus macaques have been used in mammosphere culture, and can be used as an *ex vivo* model to study breast cancer (28, 29). However, due to the low incidence of spontaneous tumors, long incubation periods, and high costs, non-human primates have not been commonly used in breast cancer research.

### Porcine BC Models

Swine have been used for decades as research subjects in diverse areas including transplantation, physiology, trauma, toxicology (49, 50), and recently cancer (30, 51–54). The porcine genome has been sequenced (55), and annotation is ongoing (56). Gene editing of pigs and creation of transgenic swine is now fairly common (52, 53, 57–60); some transgenics [such as the Oncopig (51)] are commercially available. With respect to breeding, the porcine gestation period (114 d) is relatively short (goat/sheep/cow = 150/152/283 d). In regard to pharmacokinetics, the pig has the most similarity to humans among studied mammals with respect to the cytochrome P450 enzymes (61, 62). Similar pharmacokinetic behavior between humans and pigs has been reported for a number of compounds (63), and pig is recognized as a model for enabling determinations of *in vivo* kinetics and drug metabolism in general (63, 64).

Early post-natal stem cell activity in the mammary development may be reactivated during initiation of human BC (34). RNA sequencing and immune histochemistry analysis showed that in neonatal pigs (4-39 days of age) mammary gland gene expression patterns were similar to that in human BC (34). Investigators have transformed porcine mammary epithelial cells *in vitro* with SV40 large T antigen insertion (31) or BRCA1 knockdown (65), with evidence of tumorigenicity in immunodeficient mice (31). In addition, a *BRCA1* haploinsufficient Yucatan minipig was generated with somatic cell nuclear transfer (66), but postnatal survival of cloned piglets was ≤18 days for reasons that were unclear (*BRCA1*+/– mice are phenotypically normal) (67).

As a proof of principle, our group has transformed porcine mammary epithelial cells with KRAS^G12D^ and TP53^R167H^, and produced tumors in xenografted nude mice (32). Our follow-up work in this area currently is focused on using BC relevant genes in porcine mammary epithelial cells (68–70), to be utilized in an orthotopic porcine model of TNBC. In addition, our group is endeavoring to create a transgenic, inducible, restricted model of mammary ductal neoplasia. To be clear, however, no porcine model of BC currently exists. The *KRAS/TP53* Oncopig (51) is not a BC model, but rather a “generic” porcine tumor model, potentially allowing transformation of all cell types. Of note, KRAS mutation is relatively uncommon (1-2%) in BC (68–70). Given the recent progress in porcine modeling of pancreatic cancer by multiple groups (32, 51, 54, 71–76), it is likely that the best candidate for a large animal BC model will be the pig.

### Disadvantages of Using Large Animal Cancer Models

In order to illustrate the potential disadvantages of using a large animal model of breast cancer, a comparison of porcine with murine models is described below. This does reveal a number of disadvantages with the porcine models, including:

1. **Costs**. Transgenic pigs are more expensive. Transgenic mice for BC research generally are 200-300 USD per subject (Jackson Laboratory), while transgenic pigs (e.g., the Oncopig) can be in the range of 1-2K USD. Per diem cost for pigs are generally ~10x the murine per diem at most institutions. Drug costs are higher in pigs because they require 100-1,000x the amount of drug used by mice. Labor costs are higher with pigs because of physical handling required each time a procedure is done.
2. **Husbandry**. More than 100-fold mice than pigs can be housed in the same space, which can limit experimental planning at most institutions. A Sinclair mini-pig can still reach up to 50 kg at 1.5 year, so the research facility has to accommodate animals of this size. The murine gestation period (20 d) is <20% of porcine gestation period, so crossbreeding is much quicker with mice. A relatively simple maneuver of placing a 20 g mouse under general anesthesia becomes more complicated when dealing with a 30-50 kg pig.
3. **Tools & Reagents**. The availability of antibodies, reagents, and other species-specific research tools is much greater in mice compared to pigs, though the availability for pigs has improved in the past decade.
4. **Subject Age**. In general, investigators only have access to young (<1 year) pigs; however, modeling epithelial tumors with young pigs may not be optimal.
5. **Research Community**. The number of investigators who utilize swine in cancer research is still relatively small, so other investigators may be reluctant to consider porcine experimentation.
6. **Social Issues**. Public reaction to swine use in research could be more negative compared to the reaction against rodent use. This possibility may demand more investigator effort devoted to public education.
7. **Ethical Issues**. On the surface, ethical issues with large animal research tend to be more complex than with small animal research. Among large animals, NHPs and dogs appear to be more protected by ethical standards compared to pigs in the research environment, but criteria have been difficult to quantify. In general, having strong institutional oversight of any animal research, with careful, intentional, and transparent regard for animal welfare, has been the most valuable consideration in the ethical performance of research with animal subjects.

## Other Animal Models of BC

### Overview

Rodent models of BC are available in a wide variety of genotypes and phenotypes, and have been an essential tool in preclinical BC research for decades. In order to provide historical context and relevance to the development of large animal BC models, the status of rodent BC modeling is briefly reviewed below.

### Murine BC Models

Commonly utilized murine BC models include chemically induced, cell-line derived xenograft (CDX), patient derived xenograft (PDX), humanized CDX and PDX and genetically engineered murine (GEM) models (77–80).

Polycyclic aromatic hydrocarbons such as 7,12-Dimethylbenz(a)-anthracene (DMBA) and methylcholanthrene (MC) compounds have been used in mice to induce BC (81–85). DMBA has been studied in knockout, hemizygous and SENCAR (SENsitivity to CARcinogenesis) mice, producing breast tumors after 3-34 weeks (81–84). MC has produced tumors after 7 months (85). Cell-line derived xenograft (CDX) murine models are based off the transplantation of human cell lines into immunocompromised animals (86, 87). However, a major limitation associated with these models is the lack of a functional host immune system, which means that CDX tumors do not undergo any appreciative immunoediting (88, 89). They also have reduced intra-tumoral heterogeneity which does not optimally represent a human breast tumor (86). In addition, CDX tumors are frequently derived from highly aggressive malignant tumors or pleural effusions and thus are less useful in studying the early stages of disease (86).

PDX BC models, which involve the transplantation of fragmented primary human tumor into immune deficient mice, have been shown to conserve ER, PR, and HER2 expression, particularly when grafted directly into mammary ducts (90, 91), and have shown similar metastatic progression compared to human tumors (92, 93). However, the prolonged length of time it takes to generate tumors does not always match clinical or research needs (86, 87, 94). Another concern with PDX BC modeling is that by the third *in vivo* passage murine stroma replaces human stroma, which may result in changes in paracrine regulation as well as in physical properties (95, 96). The humanized mouse model (in which an immunocompromised mouse is engrafted with components of the human immune system) has been utilized in preclinical studies on immunotherapies and BC, particularly TNBC and HER2+ cancers (97). Humanized BC models have shown clinically relevant reduction of tumor growth in response to therapy (98, 99). However, major concerns of these models include (1) a lack GM-CSF, which is important for the differentiation and maturation of the myeloid lineage; and (2) xenograft-versus-host disease, in which mature human T cells attack their murine host secondary to HLA mismatching between the hNSG and PDX components (99, 100).

There are several types of GEM models, including conventional, knockout, and conditional. Conventional GEM models are typically driven by mammary-specific promoters that direct expression of specific oncogenes (transgenes) which may not be specific to mammary epithelial cells (101–104). Expression of C-MYC, V-HRAS, WNT, PyMT, and HER2/NEU/ERBB2 through these promotes resulted in mammary tumorigenesis and metastatic lesions (105–111). Homozygous knockout of TP53 GEM mice developed lymphomas and died at around 4-6 months of age (112, 113). To overcome problem with non-specificity to certain cell lineage and prevent early embryonic death, conditional GEM models have been developed (86). WAP-Cre and MMTV-Cre mice have been able to generate hereditary breast cancer models specifically by modeling the heterozygous mutations observed in the BRCA1/BRCA2 genes (114–116). GEM models that contained a conditional mutant BRCA1 allele and a disruption in TP53 have accelerated mammary tumor development (116). TP53 in combination with other genes has been extensively studied using conditional GEM models (117).

### Rat BC Models

Rats have been considered a suitable animal model to study breast cancer due to their similarity with human mammary cancer in terms of histology, immunocytochemical markers and biological behavior of tumors (118). The histologic characteristics of normal mammary luminal epithelium and myoepithelium is similar between rats and human (119). Long term studies have shown that some rats can develop breast tumors spontaneously (118). Use of a chemical carcinogen in rats can result in a shorter latency period to tumor development (119–123). Recently, 17b-estradiol (E2) was used to induce breast tumors in August Copenhagen Irish-rats by modulating estrogen mediated mechanisms in breast cancer development (121).

In xenograft-Matrigel implantation experiments, younger rats have experienced greater tumor growth compared to older rats (124, 125). A bone metastasis immunodeficient rat model has been developed in which human breast cancer cells (MDA-MB-231) were intra-arterially injected into a hindlimb artery (126). Genetically engineered rat models of breast cancer have been developed in which HER2 and TGF-α were overexpressed through the mouse mammary tumor virus (MMTV) promoter (127). This model stochastically produced a variety of benign, hyperplastic, and malignant lesions, including ductal carcinoma *in situ* and carcinoma within a year.

In a rat model with three copies of human HRAS proto-oncogene, induction of carcinogenesis with nitrosomethylurea resulted in large mammary tumors within 8 weeks (128). Mammary carcinogenesis in rats was induced through injection of high-titer, Neu-containing, replication-defective retrovirus which produced hormonally responsive *in situ* carcinomas within 15-days post infusion, and regressed spontaneously after 20-days post infusion (129). Injection of human adenovirus type 9 (Ad9) also is known to induce estrogen-dependent mammary tumors in rats within 7-12 months (130). Overall, however, use of rats in BC research has lagged far behind the broad use of mice.

### Hamster BC Models

Similar to rats, nitrosomethylurea can induce mammary carcinoma in Syrian hamsters (*Mesocricetus auratus*), producing high-grade poorly differentiated mammary adenocarcinomas (131). Subcutaneous allogenic implantation of cell lines established from these primary tumors generated secondary tumors. Hamster models can be useful in studies on oncolytic adenoviruses, a self-replicating cancer cell-killing virus. Oncolytic adenoviruses can replicate in immunocompetent hamsters, making the hamster model of cancer a suitable non-immunocompromised model to study therapeutic potential of these viruses (132).

### Tree Shrew BC Models

The tree shrew (*Tupaia belangeri chinensis*) can develop spontaneous mammary tumors, which are similar to human papillary tumors in terms of morphology and pathology (133, 134). Chemical induction with DMBA plus medroxyprogesterone acetate (MPA) also produced breast cancer in tree shrews (135). Injection of a lentiviral vector with PyMT (polyomavirus middle T antigen, an oncogene that activates c-Src), into the mammary duct of tree shrews resulted in tumor development in all subjects by 7 weeks post-injection (136).

## Conclusions and Future Directions

The current landscape of animal modeling for breast cancer is dominated by murine models, which have developed into powerful and multi-faceted tools for the BC researcher. It would be difficult to improve on the utility that murine BC models have provided. However, there remain certain areas of research, such as device development and drug testing, which could benefit from the availability of a large animal model of BC. These BC models are still in their infancy, essentially at the point murine models were in the 1980’s. While there have been a number of principle-proving studies involving BC and large animals, a validated and tractable large animal model of BC is not yet available, necessitating that additional work needs to be done in this area if the advantages of large animal BC modeling are to be realized. While large animal BC models likely will never be able to match the proven utility and ease-of-use of murine models, the availability of validated large animal BC models could provide additional tools to the BC researcher that would address specific BC questions or BC-relevant technology development, such as those requiring a human sized subject for generation of relevant data.

## Author Contributions

All authors participated in the drafting and editing of the manuscript. All authors contributed to the article and approved the submitted version.

## Funding

This work was supported by a grant from the National Cancer Institute (R01CA222907) to MC.

## Conflict of Interest

The authors declare that the research was conducted in the absence of any commercial or financial relationships that could be construed as a potential conflict of interest.

## Publisher’s Note

All claims expressed in this article are solely those of the authors and do not necessarily represent those of their affiliated organizations, or those of the publisher, the editors and the reviewers. Any product that may be evaluated in this article, or claim that may be made by its manufacturer, is not guaranteed or endorsed by the publisher.
